# The Impact of *Hanseniaspora vineae* Fermentation and Ageing on Lees on the Terpenic Aromatic Profile of White Wines of the Albillo Variety

**DOI:** 10.3390/ijms22042195

**Published:** 2021-02-23

**Authors:** Juan Manuel Del Fresno, Carlos Escott, Iris Loira, Francisco Carrau, Rafael Cuerda, Rémi Schneider, María Antonia Bañuelos, Carmen González, José Antonio Suárez-Lepe, Antonio Morata

**Affiliations:** 1 enotecUPM, Chemistry and Food Technology Department, Escuela Técnica Superior de Ingeniería Agronómica, Alimentaria y de Biosistemas, Universidad Politécnica de Madrid, Avenida Complutense S/N, 28040 Madrid, Spain; juanmanuel.delfresno@upm.es (J.M.D.F.); carlos.escott@gmail.com (C.E.); iris.loira@upm.es (I.L.); carmen.gchamorro@upm.es (C.G.); joseantonio.suarez.lepe@upm.es (J.A.S.-L.); 2 Área Enología y Biotecnología de Fermentaciones, Facultad de Química, Universidad de la República, Gral. Flores 2124, Montevideo 11800, Uruguay; fcarrau@fq.edu.uy; 3 Comenge Bodegas y Viñedos SA, Curiel de Duero, 47316 Valladolid, Spain; cuerda@comenge.com; 4 Oenoborands SAS Parc Agropolis II-Bât 5 2196 Bd de la Lironde-CS 34603, CEDEX 05, 34397 Montpellier, France; Remi.SCHNEIDER@oenobrands.com; 5 enotecUPM, Biotechnology Department, Escuela Técnica Superior de Ingeniería Agronómica, Alimentaria y de Biosistemas, Universidad Politécnica de Madrid, Avenida Complutense S/N, 28040 Madrid, Spain; mantonia.banuelos@upm.es

**Keywords:** *Hanseniaspora vineae*, wine, non-*Saccharomyces*, fermentation, terpenes, polysaccharides

## Abstract

*Hanseniaspora vineae* is a non-*Saccharomyces* yeast that has a powerful impact on the sensory profile of wines. Its effect on the aromatic profile of non-aromatic grape varieties, such as Albillo Mayor (*Vitis vinifera*, L), during vinification is a useful biotechnology to improve sensory complexity. Fermentation in steel barrels using *Hanseniaspora vineae* and sequential inoculation with *Saccharomyces cerevisiae* have been used to study the formation of terpenes and cell lysis in the production of Albillo white wines. The GC-MS analysis profile shows a significant effect of *H. vineae* fermentation on the contents of terpenes (≈×3), mainly in linalool (>×3), β-citronellol (>×4), geraniol (>×2) and α-terpineol (≈×2). The contents of several polyoxygenated terpenes and some volatile phenols with a spicy aroma were increased during fermentation. In summary, *Hanseniaspora vineae* releases a large number of cell wall polysaccharides during fermentation that affect wine palatability and structure. *Hanseniaspora vineae* is a powerful bio-tool to enhance the fruitiness, floral notes and freshness in non-aromatic white varieties.

## 1. Introduction

The use of non-*Saccharomyces* yeasts is increasing extensively in wine biotechnology because of their ability to improve the sensory quality of wines [1,2], especially in warm areas where they can enhance the freshness of flat non-aromatic varieties [3]. *Hanseniaspora vineae* is a powerful biotechnology to improve the aroma profile of white wines through the production of aromatic compounds during fermentation [4,5,6,7]. It has been shown that the enhanced formation of 2-phenylethyl acetate [8,9] in this species is explained by the gene duplications of aromatic amino acid aminotransferases and phenylpyruvate decarboxylases [6]. Furthermore, the de novo formation of benzenoids and monoterpenes from sugars [7] has been reported in a synthetic fermentation medium. Enhanced formation of 2-phenylethyl acetate has been also reported in other *Hanseniaspora* species such as *H. osmophila* [10]; this is mainly a characteristic of *H. vineae* strains [4]. We have previously evaluated the effect of *H. vineae* in the formation of fermentative acetate esters in the neutral variety Albillo Mayor, observing an increased production of this 2-phenylethyl acetate (>30%) compared with the *S. cerevisiae* control (odour activity value 31.8) [11]. The addition of l-phenylalanine (Phe) also helps to enhance the formation of this ester [12]. The high production of floral esters is disconnected from the production of fruity esters, such as isoamyl acetate, and similar values of those compounds are as low as those formed by low producing *S. cerevisiae* strains [5]. Isoamyl acetate produces banana smells and it is correlated with aroma standardisation in white wines. Non-*Saccharomyces* yeasts do not only have applications in alcoholic fermentation; they can also be used in the ageing on lees technique. This technique consists of maintaining contact between the lees and the wine. During these periods, cellular autolysis of the yeast takes place, causing the release of cellular compounds into the wine [13]. Among the compounds released, the cell wall polysaccharides have special importance: these compounds modify the organoleptic characteristics of aged wine. It is known that not all yeast species release polysaccharides at the same rate, so a technique to accelerate the ageing on lees process includes the use of yeasts selected for their high polysaccharide release capacity [14]. In this regard, *H. vineae* also produces a mouth effect perceptible in tasting, thus increasing roundness and body and improving the structure of white wines [8]. A high or similar absorbance at 260 nm and 280 nm with other yeast species has been observed, with the high release of cell wall polysaccharides [5] related to the contents of nucleic acids and proteins that can be correlated with the cell autolysis [15,16,17]. The release of cell wall polysaccharides during yeast autolysis by H. vineae has been similar to other *S. cerevisiae* strains with high production of these polymers, but lower than other highly polysaccharide-producing species such as *Schizosaccharomyces pombe* [5,17,18].

Due to its increased impact in the Ribera de Duero wine region of Spain, the Albillo Mayor (*Vitis vinifera* L.) variety has been used to make white wines, usually barrel-aged with or without on-lees processes. Although there might be different Albillo grapes in Spain (Albillo Real, Mayor, or Dorado), it is a suitable variety for ageing with good structure and body but a flat aromatic profile with low contents of fruity and floral varietal aromas [19].

The main goal of this research is to assess the formation of terpenes and other phenolic flavours during fermentation of the neutral variety Albillo (*V. vinifera* L.) by *Hanseniaspora vineae*, and the effect in the release of polysaccharides that can improve body and structure in white varieties.

## 2. Results

### 2.1. Morphology and Molecular Identification of H. vineae

**Figure 1:**
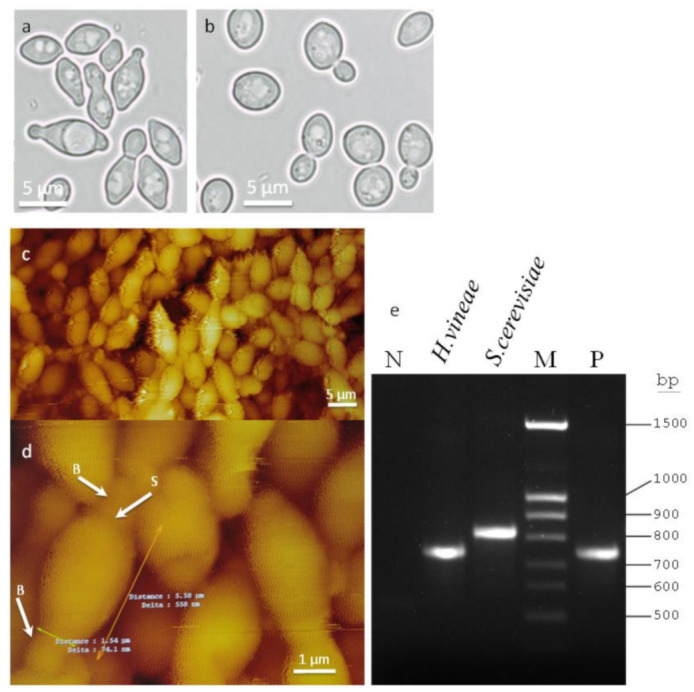
Optical microscopy of *Hanseniaspora vineae* (**a**) and *Saccharomyces cerevisiae*. (**b**) Atomic force microscopy (AFM) 3D topographic images of lyophilised cells of *Hanseniaspora vineae* with pseudocolour scale representation (dark areas represent lower height values while brighter areas represent higher height values). (**c**) A 50-micron scan size image showing typical apiculate morphology with a lemon-like shape and bipolar budding. (**d**) A 13.5-micron scan size image showing details of the cells. Thin arrows show typical dimensions of the cell and bud length. Thick white arrows labelled as “B” indicate the positions of the buds. The thick white arrow labelled as “S” indicates the position of the scar. (**e**) Agarose gel electrophoresis of a PCR product obtained from the amplification of the 5.8S-ITS (Internal Transcribed Spacer) region using the universal primers ITS1–ITS4 from *S. cerevisiae* and *H. vineae*; M, molecular weight marker (100-bp DNA ladder). N: negative control without DNA in the PCR reaction; P: positive control with purified genomic DNA of *Hanseniaspora* as a template.

Optical microscopy was used to show the special geometry of *H. vineae* (Figure 1a) compared to the typical ellipsoidal shape of *Saccharomyces cerevisiae* (Figure 1b). Additionally, the bipolar budding of *H. vineae* can be observed compared to the multipolar budding in *S. cerevisiae*. Several details on the development of new buds can be observed, from early, low-sized stages to the later, larger stages with a clearly defined septum. Lyophilised cells of *H. vineae* were scanned by atomic force microscopy (AFM) in resonant mode using two scanning frameworks: 50 µm (Figure 1c) and 13.5 µm (Figure 1d). AFM was used to show better 3D details of cell geometry, bud location and bud scars. The typical apiculate shape (lemon-like) of the species *Hanseniaspora* can be observed, with bipolar budding (Figure 1c). In Figure 1d, the size of the yeasts and buds can be observed in detail, as well as the diameter of the bud scars. The bud grows until it has a suitable size; later, it is separated. After the separation, the joining region shows a ring scar. Several rings in the older cells can be observed, indicating the number of budding processes in the same pole during the multiplication phase. Strong adhesion was present between the AFM tip and the cell membrane, especially on the deeper parts (darker areas). This can be appreciated in some areas of the images as vertical fuzzy lines. These lines are artefacts due to unstable oscillation of the tip on top of these areas and are not related to the morphology of the cell.

Molecular characterisation of *H. vineae* can be performed by PCR amplification of the 5.8S-ITS (Internal Transcribed Spacer) region followed by agarose gel electrophoresis of the fragments (Figure 1c). PCR and supplementary sequencing of the fragment were used to ensure the identification of *H. vineae*. Using ITS1-ITS4 primers *H. vineae* can be clearly distinguished from *S. cerevisiae* with a fragment at 750 bp. The ITS fragment was sequenced, and alignments were compared with other species: *Saccharomyces cerevisiae*, *Hanseniaspora uvarum,* and *Schizosaccharomyces pombe* (Appendix A.

### 2.2. General Oenological Parameters

The grape must used had a density of 1086 g/L (203 g/L sugars), a pH of 3.16, and total acidity of 7.5 g/L expressed as tartaric acid. After fermentation, wines sequentially fermented with *H. vineae*/*S. cerevisiae* and *S. cerevisiae* controls both had an alcoholic degree of 11.9% vol. and a pH of 3.2. However, the total acidity was slightly higher in *S. cerevisiae* (6.3 ± 0.1 g/L) compared with sequential fermentation with *H. vineae* (5.8 ± 0.2 g/L). Both showed a moderate volatile acidity, 0.45 ± 0.07 g/L expressed as acetic acid in the control fermentation by *S. cerevisiae*, and 0.36 ± 0.02 g/L in the case of *H. vineae*. These values are typical of the wines of this variety.

### 2.3. Aroma Compounds

The analysis of aroma compounds focused on terpenes, due to the neutral aromatic profile of the Albillo variety, to evaluate the effect of *H. vineae* in the formation of these compounds. Other compounds were also analysed with herbal, medicinal, floral, or spicy scents.

#### 2.3.1. Terpenes and Polyoxygenated Terpenes

The concentration of total terpenes was approximately three times higher in sequential fermentations with *H. vineae* compared with the control fermentation by *S. cerevisiae* (Table 1). Significant differences were found in linalool, terpinen-4-ol, β-citronellol, geraniol and α-terpineol. The amounts of all of them were two to four times higher than in the control fermentation by *S. cerevisiae* (Table 1).

The concentration of oxygenated terpenes was more variable, but the global amount was slightly higher in *H. vineae* sequential fermentations (×1.2, Table 1). Furanic linalool oxides and cis-pyran linalool oxide had significantly higher concentrations with *S. cerevisiae* but the remaining polyoxygenated terpenes were formed in significantly higher concentrations during the sequential fermentations by *H. vineae* (Table 1).

#### 2.3.2. Aldehydes, C6 Compounds, Alcohols, and Volatile Phenols

Sequential fermentations of *H. vineae* significantly increased the contents in aldehydes, alcohols and volatile phenols (Table 2). Concentrations of 2-hexenal were increased ×8.9, total alcohols ×3, and spicy volatile phenols ×4–×10 (Table 2). These compounds were studied because of their interesting sensory contribution to the wine aroma of neutral varieties.

### 2.4. Total Polyphenol Index and Chromatic Characteristics

**Figure 2:**
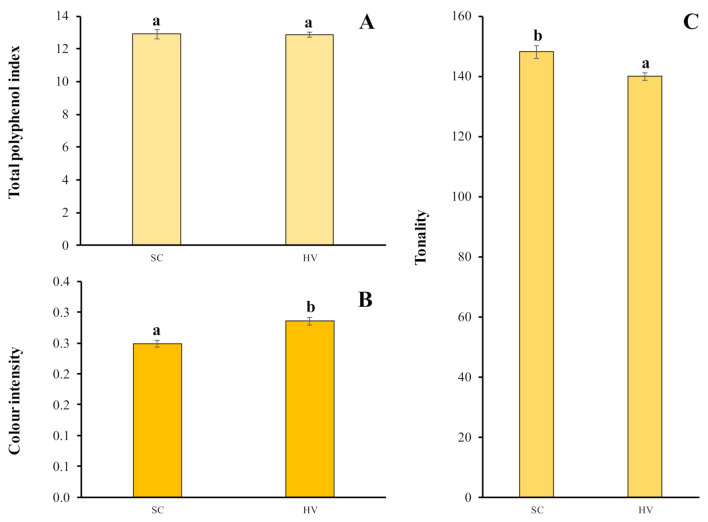
Total polyphenol index (absorbance units) (**A**), colour intensity (absorbance units) (**B**), and tonality (dimensionless) (**C**). Mean ± SD for three replicates. Bars with the same letter are not significantly different (*p* < 0.05).

Figure 2 shows the total polyphenol index (TPI) and the colour parameters obtained by measuring absorbance at 280, 420, 520 and 620 nm. No significant differences were found in the total polyphenol index (TPI) for the two yeast species studied. All the values obtained were between 12.6 and 13.2.

Colour intensity values of around 0.23 absorbance units were determined in *H. vineae* wines. These results were statistically higher than those obtained in *S. cerevisiae* wines. In contrast to these results, the *S. cerevisiae* samples showed a higher absorbance at 420 nm and, therefore, a high tonality compared to *H. vineae* wines. 

### 2.5. Polysaccharides Content

**Figure 3:**
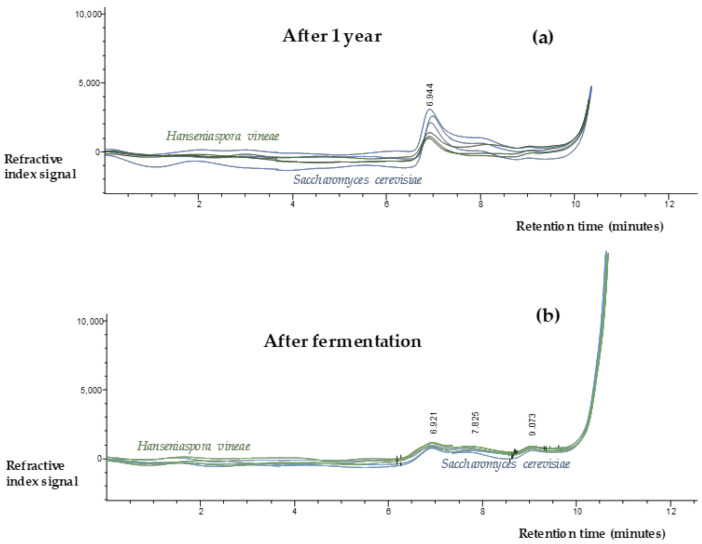
Molecular exclusion Liquid Chromatography-Refractive Index Detection (LC-RID) chromatograms of cell wall polysaccharides in 120 L triplicate fermentations of *S. cerevisiae* and *H. vineae* (**a**). After one year of ageing on lees (**b**).

Table 3 shows the results of cell wall polysaccharides measured by molecular exclusion Liquid Chromatography-Refractive Index Detection (LC-RID). In the group of wines analysed after the end of the alcoholic fermentation, polysaccharide concentrations of around 200 mg/L could be observed without significant differences between the two species of yeast studied. Regarding wines aged on lees, the ones aged in the presence of *S. cerevisiae* biomass showed polysaccharide values of around 380 mg/L. This concentration was significantly higher than that found in wines aged on *H. vineae* lees with a polysaccharide concentration of 180 mg/L. Although there is a lower concentration of polysaccharides in *H. vineae* treatments, it is interesting that they are compounds of higher molecular size (Figure 3) that might have a positive effect on the final perception of the wine. 

## 3. Discussion

### 3.1. General Oenological Parameters

General oenological parameters showed typical values for the Albillo Mayor variety and in many white wines. It should be noted that the moderate alcoholic degree and the suitable acidity will produce a balanced taste, however, independent of the yeast species effect. It is noticeable that fermentations by *H. vineae* produced a slightly lower volatile acidity, a typical characteristic of this species compared to other *Hanseniaspora* species [4].

### 3.2. Terpenes and Polyoxygenated Terpenes

The effect in aroma due to the formation of acetate esters and benzenoids by *H. vineae* during fermentation has been analysed in several articles [4,5,6,7,8,9]. However, the effect on varietal terpenes has been less analysed and their formation or release from precursors have a strong impact on the floral and citric aroma of wines. The most important terpenes in Muscat and relative varieties of wines are linalool, geraniol, nerol, α-terpineol and hotrienol [23]. In neutral varieties such as Albillo, the contents are very low and frequently below or around the odour thresholds. Terpenes were highly increased by the fermentations of *H. vineae*; several of them with concentrations higher than the sensory threshold, especially for floral/citric terpenes. Linalool was below the sensory threshold in fermentations with *S. cerevisiae* and perceptible in the fermentations of *H. vineae* with an odour activity value (OAV) of 2.8. Concentrations of linalool in wines ranged from 6 to 375 µg/L [23], 30 to 356 µg/L in Muscat wines and 6 to 58 µg/L in other varieties [24]; therefore, during fermentation *H. vineae* produces terpene contents close to Muscat varieties. The synthesis de novo of monoterpenes by *H. vineae* was proved in a synthetic fermentation medium not containing monoterpene precursors, as it was reported recently [4], and in a smaller proportion in *Saccharomyces* strains [29]. β-citronellol was approximately at the sensory threshold when Albillo musts were fermented by *S. cerevisiae* (OAV 1.3) but at much higher concentrations in the *H. vineae* fermentations (OAV 5.7). Concentrations of β-citronellol in Muscat wines ranged from 3 to 25 µg/L [24]. Geraniol, a floral terpene with a rose perception, was slightly above the sensory threshold in the *S. cerevisiae* fermentations (OAV 1.7) and much more intense with *H. vineae* (OAV 4.9). Concentrations of geraniol in wines ranged from 14 to 187 µg/L [23], and 5 to 60 µg/L in Muscat wines [24]. Previous values highlight the high impact of *H. vineae* on the formation of fruity and floral terpenes as β-citronellol and geraniol. Sweet and balsamic terpineol isomers were also significantly increased by *H. vineae* but without a clear impact on the aroma because of their low concentrations compared with their odour threshold. Therefore, the impact of *H. vineae* on the aroma is more intense through the production of floral and citric terpenes. The wine aroma is scented with floral, lemon, citric and rose nuances when it is fermented by *H. vineae* compared with the flat and neutral sensory profile in the controls fermented by *S. cerevisiae*. 

Polyoxygenated terpenes were in similar concentrations in sequential fermentations by *H. vineae* and control fermentations with *S. cerevisiae*. However, significantly higher production of some of these compounds is found in *H. vineae* treatments compared to pure *S. cerevisiae* fermentations. Moreover, and even if it is difficult to find data on some of these terpene derivatives, their sensory thresholds are much higher than normal terpenes, ranging 3–5 mg/L for linalool oxides (Table 1) [23,24]. Therefore, the concentrations in both sequential and control fermentations are quite below the odour threshold. 

### 3.3. Aldehydes, C6 Compounds, Alcohols and Volatile Phenols

Most of these compounds, even with interesting sensory profiles, were below the corresponding odour thresholds (Table 2). However, the production of 2-hexenal was strongly increased with an average OAV of 5.3 in control fermentations and 47 in the sequential fermentations with *H. vineae*. The formation of 2-phenylethyl and benzyl alcohol was also significantly increased in *H. vineae* treatments; however, the concentration was just below the odour threshold so probably without impact in the sensory profile. *H. vineae* has been described as a powerful species to synthesise the formation of these two phenolic alcohols and its corresponding acetate ester [4,5,6,7].

Lastly, it is interesting to note that the formation of spicy volatile phenols with clove aroma (eugenol and isoeugenol) is enhanced in the fermentations with *H. vineae* compared with the control (Table 2). In eugenol, concentrations are below the odour threshold, but for isoeugenol the OAV is 4. Therefore, *H. vineae* also favours the development of spicy nuances during fermentation that might contribute to the increase in sensory complexity. The clove smell produced by *H. vineae* can help to better integrate the ageing aroma when using oak barrels or ageing on lees.

### 3.4. Total Polyphenol Index and Chromatic Characteristics

The yeast species did not influence the TPI of the wines after fermentation. The values obtained for this parameter are typical of young white wines, and similar results were reported by other authors [30].

The colour intensity includes the sum of the absorbance at 420 nm (yellow), 520 nm (red) and 620 nm (blue). It is noted that the highest colour intensity is seen in wines fermented by *H. vineae*. These results are slightly higher than those shown [31] in white wines of Emir, Chardonnay and Muscat grape varieties. However, this was not due to an increase in the absorption in the yellow component that occurs in oxidised wines, since the highest tonality values were obtained in wines fermented by *S. cerevisiae*. This decrease in tonality is of oenological interest because low levels of tonality are identified with young pale wines.

### 3.5. Polysaccharides Content

Wine polysaccharides are the main macromolecules of colloidal nature; these compounds have a major impact on wine sensory perception. Polysaccharides increase the density and body of the wine, modulating the astringency perception. In addition, polysaccharides are able to interact with volatile compounds [32].

The origin of this large family of compounds is the cell wall of the grape and yeasts. For this reason, the yeast strain used for both fermentation and wine ageing is of major importance. Figure 3 shows the chromatograms of the cell wall polysaccharides measured by molecular exclusion LC-RID. In this figure, it can be seen that the peak of the wines after one year of ageing on lees is bigger than the peak of the wines after fermentation; besides, this peak is clearly defined in the retention times of the calibrated pullulans (6–10 min). It is important to note that all the wine samples had a high concentration of polysaccharides, above 100 mg/L. This is because the measurements include both grape and yeast cell wall polysaccharides.

In relation to wines after fermentation, the release of polysaccharides was similar in both yeast species. Therefore, *Hanseniaspora* could be an interesting non-*Saccharomyces* yeast to replace traditional fermentations without affecting the polysaccharide content. These results are in contrast to those obtained by [33], who observed higher polysaccharide contents in pure fermentation of *Hanseniaspora* than in *Saccharomyces* fermentations.

After long periods of ageing on lees, the release of polysaccharides from the cell wall of *H. vineae* is lower than that of the *S. cerevisiae* we selected for its fast autolysis. In previous investigations, we have monitored a similar release of polysaccharides from the exogenous addition of *H. vineae* compared to two selected *S. cerevisiae* strains [5], probably because a different lysis process was used. Therefore, it is necessary to carry out studies to find out the nature of the *H. vineae* polysaccharides, as well as their polysaccharide transfer kinetics during cellular autolysis.

## 4. Materials and Methods

### 4.1. Yeasts and Inoculations

The fermentations in this study were made with an *H. vineae* yeast, a strain that was isolated by Professor Francisco Carrau (Facultad de Química, Universidad de la República, Montevideo, Uruguay). The yeast strain Fermivin 3C (*S. cerevisiae*) used as a control in this study is a selected commercial yeast strain (Oenobrands SAS, Montpellier, France). *H. vineae* yeast was grown in liquid YPD media and inoculated at a population of 7-log CFU/mL. The *Saccharomyces* 3C was inoculated at the same population as active dried yeast. 

### 4.2. Atomic Force Microscopy

A 10 µL drop of suspension in sterile water of *H. vineae* cells growth in YPD-agar was frozen at −80 °C in a CO_2_ freezer on a coverslip. After that, the sample was lyophilised in a freeze-drying device.

Topographic measurements of the cells were carried out using a Nano-Observer AFM (Concept Scientific Instruments, Les ULIS, France), operating in resonant mode. A 1 N/m rectangular silicon cantilever (model Fort, AppNano, Mountain View, CA, USA) with an 8 nm nominal tip radius was selected. Typical setpoint amplitudes of 4–5 volts were used during the measurements with high values of feedback Proportional and Integral gains (P and I) to compensate for the high topographic variations (1–4 microns). 

### 4.3. PCR Analysis of the rDNA–ITS Region

Cells were directly collected from a fresh yeast colony and suspended in 100 µL distilled water. Cell suspensions were heated at 94 °C for 5 min, vortexed, and 2 µL directly used for DNA amplification. The ITS region was amplified using the primers ITS1 (5′ TCCGTAGGTGAACCTGCGG 3′) and ITS4 (5′ TCCTCCGCTTATTGATATGC 3′) according to [34]. The PCR reaction mix contained 0.5 μM of each primer, 200 μM deoxynucleotides, 1.5 mM MgCl_2_ and 1 × buffer (Takara, Shiga, Japan). The suspension was heated at 94 °C for 4 min, and one unit of the Taq Polymerase (Takara) was then added to each tube. PCR conditions were as follows: 35 cycles of denaturing at 94 °C for 1 min; annealing at 55 °C for 1 min and extension at 72 °C for 1 min; a final extension at 72° for 7 min. All reactions were repeated at least twice, always including both negative (DNA-free) and positive controls. The PCR products were separated on 1.6% agarose gel, stained with SYBR™ Safe stain (Invitrogen, Thermofisher, Madrid, Spain) and visualised under blue-light transilluminator. Sizes were determined by comparison against the DNA length standard (100 bp DNA ladder, Invitrogen). PCR fragment corresponding to *Hanseniaspora vineae* was sequenced (Secugen, Madrid, Spain) and used for identification by comparison with other species.

### 4.4. Musts and Fermentations

The must is from the Albillo variety (*Vitis vinifera* L.) from the Ribera de Duero region in Spain. Albillo is a neutral variety with low contents of aromatic compounds. The grapes were pressed in a pneumatic press at less than 2 bar and subsequently settled at a low temperature (8 °C) for 24 h. Musts were fermented at Comenge Cellars (Curiel de Duero, Valladolid, Spain) in 120 L stainless steel barrels in triplicate at room temperature (18 °C). The evolution of the fermentation process lasted a month and was monitored by measuring density and temperature. The samples were taken once at the end of the fermentation and stored in bottles at 4 °C.

### 4.5. Oenological Parameters Analysis

Ethanol (% *v*/*v*), pH, total acidity (g/L) expressed as tartaric acid, volatile acidity (g/L) expressed as acetic acid, and glucose/fructose content (g/L) were analysed by Fourier transform infrared spectroscopy (FTIR), using an OenoFoss instrument (FOSS Iberia, Barcelona, Spain). This method allows the quick attainment of a large number of oenological parameters [35], and the integrated software of the OenoFoss machine provides these parameters directly.

### 4.6. Terpenes and Aroma Composition by GC-MS

One hundred millilitres of centrifuged grape juice (15 min at 6.000 rpm) was added with 100 µL of 2-octanol (as IS) and purified in an SPE cartridge (Solid Phase Extraction) Bond Elut ENV of 500 mg and 6 mL (Agilent Technologies, Santa Clara, CA, USA) [36]. Cartridges were previously conditioned by sequentially washing with 5 mL of methanol, 5 mL of hydroalcoholic solution (12% *v*/*v*) and 5 mL of water. Compounds were eluted with P/D (pentane-dichloromethane, 50/50), dried, and finally re-dissolved in P/D up to a final volume of 200 μL. A gas chromatograph 7890A with a mass spectrometer 5975C inert MSD (Agilent Technologies) was used. A constant flow of 2.1 mL/min of He was used as the carrier gas. The injected volume was 5 μL in splitless mode, with 17.33 psi pressure (septum purge flow 15 mL/min and splitless time 1 min). A DB-WAX IU column (60 m × 0.25 mm × 0.25 μm) was used. The injector temperature was maintained at 180 °C for 1 min and later heated up to 260 °C at 250 °C/min. The oven was maintained at 60 °C for 15 min and then increased up to 220 °C (at 3 °C/min) for 25 min. The fragmentation voltage was 70 eV. MS was recorded in scan mode (*m*/*z* 10−1000). The compounds were identified by their m/z fragments and retention times, compared with standards. The quantification was carried out using the method of internal standard patterns.

### 4.7. Total Polyphenol Index and Chromatic Characteristics

The total polyphenol index (TPI) was obtained by measuring absorbance at 280 nm with a 1 cm path length quartz cell, after a previous dilution of 10 volumes [37]. Colour intensity and tonality were measured by determining absorbance at 420, 520 and 620 nm with a 1 mm path length glass cell according to the procedure described by [38]. 

The absorbance measurements were performed using an 8453 spectrophotometer from Agilent Technologies™ (Palo Alto, CA, USA). All the samples were previously centrifuged at 1200 rcf for 3 min.

### 4.8. Polysaccharides by HPLC-RI

The polysaccharide content was measured in two groups of samples in triplicate. The first group included wines after fermentation with *Saccharomyces cerevisiae* and *Hanseniaspora vineae*, independently. The other group of samples analysed was wines after one year of ageing on lees. The yeast biomasses used for this ageing were lees of *S. cerevisiae* and *H. vineae*, separately.

The polysaccharide content was determined by an HPLC-IR technique, according to the method described by [39]. An 1100 HPLC chromatograph (Agilent Technologies, Palo Alto, CA, USA) equipped with a refractive index detector with Ultrahydrogel 250 molecular exclusion column (Waters, Milford, MA, USA) was used. A calibration curve was constructed from the following pullulan standards (polymaltotriose) (Shodex, Showa Denko K.K, Japan) to determine the concentration of polysaccharides in the samples: P-800 (788 kDa), P-400 (404 kDa), P-200 (212 kDa), P-100 (112 kDa), P-50 (47.3 kDa), P-20 (22.8 kDa), P-10 (11.8 kDa) and P-5 (5.9 kDa). The polysaccharide content (mg/L) was obtained after integration of the chromatogram peak areas. This integration value was fed into the pullulans calibration curve to obtain the polysaccharides concentration value. 

## 5. Conclusions

*H. vineae* is a powerful bio-tool able to transform a neutral variety, such as Albillo, with low floral and citric fruit into a quasi-terpenic variety reaching a global content of higher than 300 µg/L, but at the same time with three of them showing OAV in the range of 2.8–5.7. Some spicy nuances are also enhanced by the formation of volatile phenols. Additionally, the effect on mouth palatability is quite fast, with an intense release of cell wall polysaccharides during fermentation. The evolution of these polysaccharides in time is at consistently lower contents than *S. cerevisiae* but with a high molecular size that might contribute to a better mouth perception. 

## Figures and Tables

**Table 1:** Concentrations of terpenes and polyoxygenated terpenes (µg/L) in wines fermented by *Saccharomyces cerevisiae* and by *Hanseniaspora vineae* followed sequentially by *S. cerevisiae*. Values are means ± standard deviations (*n* = 3). A different letter in the same row means significant differences (*p* < 0.05). Values in bold type are those with high differences and above the odour threshold in wines.

Type	Compound	Odour Threshold	Odour	*Saccharomyces cerevisiae*	*Hanseniaspora vineae* Followed Sequentially by *S. cerevisiae*
Monoterpenes	Linalool	25 a	Floral, lemon	18.0 ± 3.8 ^a^	**69.9 ± 16.1 ^b^**
	terpinen-4-ol	250 c	Pine	15.8 ± 2.5 ^a^	20.7 ± 3.6 ^b^
	Epoxylinalool	-	-	18.4 ± 1.4 ^a^	18.3 ± 1.9 ^a^
	β-citronellol	18 b	Citric	23.6 ± 2.0 ^a^	**103.0 ± 7.9 ^b^**
	Geraniol	20 a	Rose	35.0 ± 2.3 ^a^	**98.1 ± 10.1 ^b^**
	α-terpineol	250 c	Pleasant, sweet	3.2 ± 0.6 ^a^	6.1 ± 1.3 ^b^
	Totals			114.0 ± 12.5 ^a^	316.1 ± 41.0 ^b^
Polyoxygenated terpenes	cis-furan-linalool oxide	3000–4000 d	Earthy, leafy	16.6 ± 1.4 ^b^	8.6 ± 1.4 ^a^
	trans-furan-linalool oxide	3000–4000 d	Earthy, leafy	20.7 ± 3.8 ^b^	10.5 ± 2.0 ^a^
	cis-pyran linalool oxide	3000–5000 e	-	55.2 ± 7.0 ^b^	22.9 ± 5.4 ^a^
	trans-pyran linalool oxide	3000–5000 e	-	24.1 ± 1.6 ^a^	57.6 ± 4.7 ^b^
	2,6-dimethyl-3,7-octadiene-2,6-diol	-	-	4.7 ± 1.6 ^a^	9.4 ± 3.1 ^b^
	2,6-dimethyl-1,7-octadiene-3,6-diol	-	-	5.8 ± 1.9 ^a^	16.0 ± 3.2 ^b^
	3,7-dimethyl-1,7-octanediol	-	-	4.0 ± 1.6 ^a^	11.4 ± 1.6 ^b^
	8-hydroxylinalool	-	-	25.5 ± 5.0 ^a^	51.2 ± 5.4 ^b^
	Totals			156.7 ± 23.9 ^a^	187.6 ± 26.8 ^b^

References for odour thresholds: a [20], b [21], c [22], d [23], e [24].

**Table 2:** Concentrations of aldehydes, C6 compounds, alcohols and volatile phenols (µg/L) in wines fermented by *Saccharomyces cerevisiae* by *Hanseniaspora vineae* followed sequentially by *S. cerevisiae*. Values are means ± standard deviations (*n* = 3). A different letter in the same row means significant differences (*p* < 0.05). Values in bold type are those with high differences and above the odour threshold in wines.

Type	Compound	Odour Threshold	Odour	*Saccharomyces cerevisiae*	*Hanseniaspora vineae* Followed Sequentially by *S. cerevisiae*
Aldehydes	Benzaldehyde	2000 a	Almonds	4.2 ± 1.4 ^a^	17.8 ± 3.0 ^b^
	Trans-2-hexenal	17 b	Green, apple	**90.0 ± 10.0 ^a^**	**799.1 ± 97.2 ^b^**
	Totals			94.2 ± 11.4 ^a^	816.9 ± 100.2 ^b^
C6 compounds	trans-3-hexen-1-ol	400 c	Green	178.6 ± 14.2 ^b^	102.8 ± 12.0 ^a^
	cis-2-hexen-1-ol	400 d	-	6.9 ± 3.2 ^a^	58.0 ± 12.8 ^b^
	cis-3-hexen-1-ol	400 c	Green	5.7 ± 1.0 ^a^	6.2 ± 1.3 ^a^
	1-hexanol	8000 e	Grass	450.4 ± 27.4 ^b^	248.1 ± 25.6 ^a^
	Totals			641.5 ± 45.9 ^b^	415.2 ± 51.8 ^a^
Alcohols	1-octanol	120 e	Waxy, green, citrus	6.2 ± 2.6 ^a^	13.4 ± 2.1 ^b^
	1-octen-3-ol	-	-	22.0 ± 5.2 ^a^	92.1 ± 9.1 ^b^
	benzyl alcohol	200,000 b	Chemical, fruity	28.0 ± 4.4 ^a^	43.0 ± 3.7 ^b^
	Phenylethyl alcohol	14,000 a	Rose petals	4675.7 ± 976.3 ^a^	13,871.5 ± 1002.4 ^b^
	Totals			4731.9 ± 988.4 ^a^	14,020.1 ± 1017.3 ^b^
Volatile phenols	Eugenol	6 a	Clove, spicy	0.4 ± 0.2 ^a^	4.3 ± 1.1 ^b^
	Isoeugenol	6 a	Clove, spicy	6.2 ± 1.1 ^a^	**24.3 ± 3.1 ^b^**
	methyl salicylate	-	Sweet	0.0 ± 0.0 ^a^	0.0 ± 0.0 ^a^
	ethyl salicylate	-	Sweet	0.0 ± 0.0 ^a^	0.0 ± 0.0 ^a^
	Totals			6.7 ± 1.3 ^a^	28.5 ± 4.2 b^b^

References for odour thresholds: a [25], b [26], c [20], d [27], e [28].

**Table 3:** Polysaccharides (mg/L) measured by HPLC-RI. Means ± standard deviation of three replicates. Values with the same letter are not significantly different (*p* < 0.05).

		Polysaccharides Content (mg/L)
Wines fermented	by *Saccharomyces cerevisiae*	194.15 ± 16.20 ^a^
	by *Hanseniaspora vineae* followed sequentially by *S. cerevisiae*	203.97 ± 21.11 ^a^
Wines aged on lees (1 year)	*Saccharomyces cerevisiae* lees	380.22 ± 16.20 ^b^
	*Hanseniaspora vineae* lees	183.99 ± 21.11 ^a^

**Figure A1:**
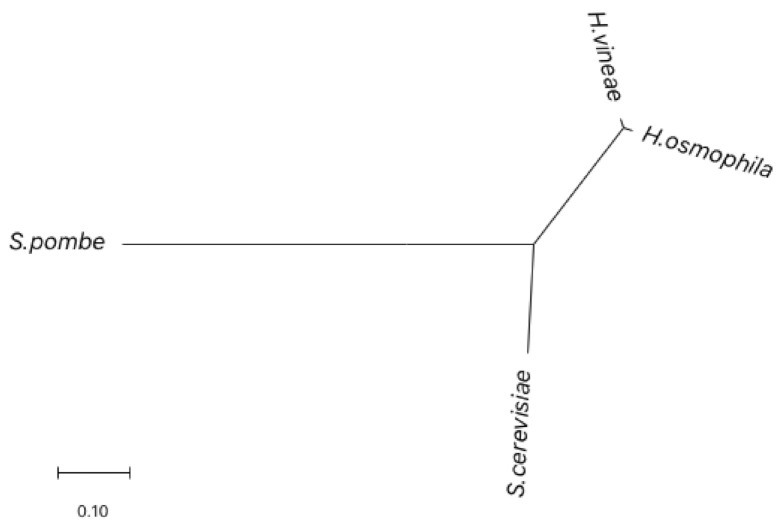
Phylogenetic relationships among wine yeast species based on analysis of ITS gene sequences.

**Figure A2:**
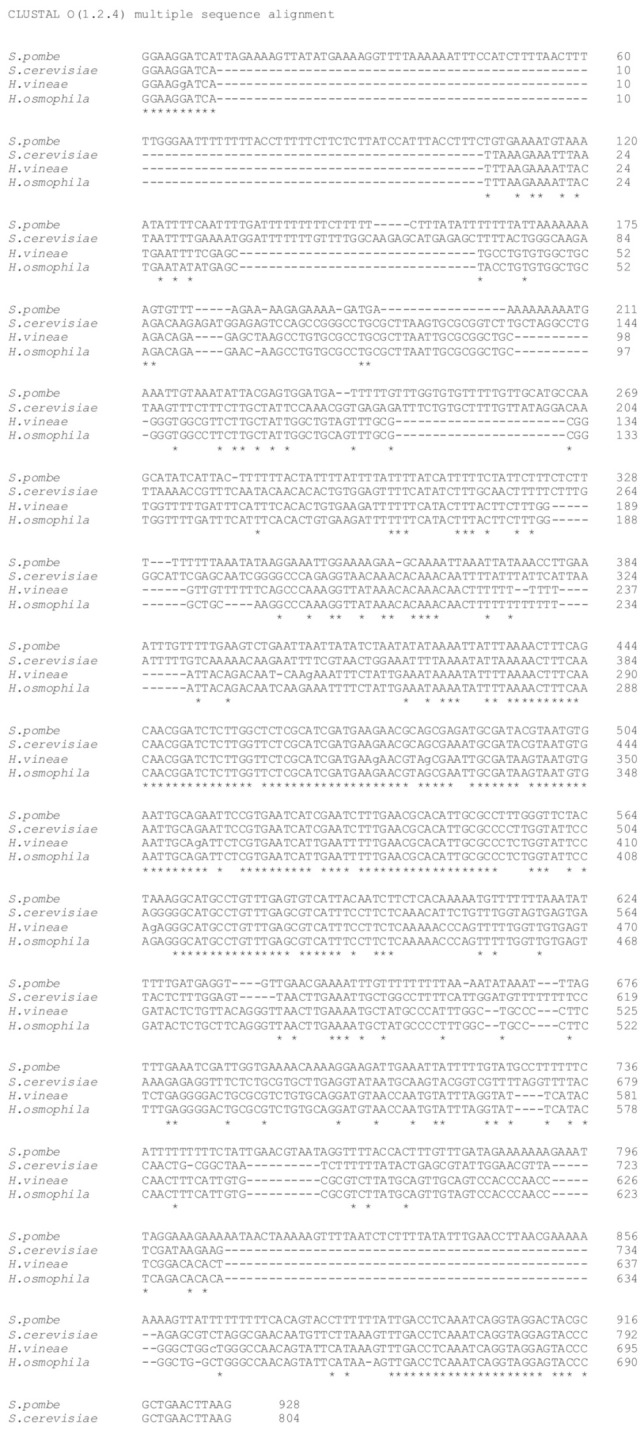
Multiple alignments of complete 5.8S rDNA sequence and Internal Transcribed Spacer 1 and 2 sequences, for: *Schizosaccharomyces pombe*, *Saccharomyces cerevisiae*, *Hanseniaspora vineae* and *Hanseniaspora osmophil*a.

## Appendix A

Phylogenetic relationships among wine yeast species based on analysis of ITS gene sequences (Figure A1). The evolutionary history was inferred by using the Maximum Likelihood method based on the Tamura–Nei model in MEGA X software. *Schizosaccharomyces pombe* was used as the outgroup. GenBank accession numbers follow strain numbers: *Saccharomyces cerevisiae* KT958553; *Hanseniaspora vineae* used in this study; *Hanseniaspora osmophila* KY103543; *Schizosaccharomyces pombe* EU916982.

Multiple alignments of complete 5.8S rDNA sequence and Internal Transcribed Spacer 1 and 2 sequences. The ITS sequences were analysed and aligned using BLAST and CLUSTAL O software (Figure A2). GenBank accession numbers follow strain numbers: *Saccharomyces cerevisiae* KT958553; *Hanseniaspora vineae* used in this study; *Hanseniaspora osmophila* KY103543; *Schizosaccharomyces pombe* EU916982.
